# The Association of Race With Outcomes in Hospitalised Patients With Hepatorenal Syndrome: Nationwide Cohort Study

**DOI:** 10.1111/liv.16226

**Published:** 2024-12-25

**Authors:** Shahana Prakash, Mark Vander Weg, Tomohiro Tanaka

**Affiliations:** ^1^ Department of Internal Medicine University of Iowa Carver College of Medicine Iowa City Iowa USA; ^2^ Department of Community and Behavioral Health University of Iowa College of Public Health Iowa City Iowa USA; ^3^ Division of Gastroenterology‐Hepatology, Department of Internal Medicine University of Iowa Carver College of Medicine Iowa City Iowa USA; ^4^ Department of Health Management and Policy University of Iowa College of Public Health Iowa City Iowa USA

**Keywords:** acute kidney injury, hepatorenal syndrome, HIspanic paradox, Mediation analysis, racial disparity

## Abstract

**Introduction:**

Racial/ethnic disparities have been previously reported in renal and hepatic disease care; however, acute kidney injury (AKI) in the setting of cirrhosis (hepatorenal syndrome [HRS]‐AKI) despite its complexity requiring a multidisciplinary approach, remains understudied.

**Methods:**

To identify unique associations of clinical and sociodemographic factors with mortality and length of stay (LOS) among patients hospitalised with HRS‐AKI, hierarchical regression analysis was conducted, along with a mediation analysis to estimate how race‐related differences in in‐hospital mortality were influenced by payer type, area household income, and clinical severity.

**Results:**

Black patients demonstrated a significantly higher odds of in‐hospital mortality, compared to their white counterparts, adjusting for (1) sex and age, (2) sex, age, payer type, and area household income and (3) sex, age, and clinical severity [OR 1.16–1.20, 95% confidence intervals (CI) > 1]. Higher mortality rates among Black patients were partially mediated by clinical severity and area household income [proportion mediated (PM): _0.189_0.19_0.192_ and _0.16_0.17_0.18_, respectively]. Black patients with HRS‐AKI had longer LOS than White patients. Hispanic patients tended to have lower odds of in‐hospital mortality [OR: _0.77_0.86_0.97_] despite their lower income and more severe illness.

**Conclusion:**

Our nationwide US study demonstrated that, partly due to higher clinical severity and lower household income, Black patients with HRS‐AKI experience higher inpatient mortality, compared to White patients. On the other hand, Hispanics with HRS‐AKI have a survival advantage. More awareness is warranted to address racial disparities in HRS‐AKI outcomes.

**Abbreviations**
AKI: Acute kidney injury
APR‐DRG: All Patient Refined Diagnosis Related Group
CI: confidence interval
CKD: chronic kidney disease
eGFR: estimated glomerular filtration rate
HCUP: Healthcare Cost and Utilisation Project
HRS‐AKI: hepatorenal syndrome–acute kidney injury
ICD‐10: International Classification of Diseases, 10th Revision
LOS: length of stay
LT: liver transplantation
NIS: National Inpatient Sample
RR: risk ratio
SD: standard deviation
SDDOH: structural and social determinants of health
SPB: spontaneous bacterial peritonitis
US: United States

**Summary**

- This study examined differences in hospital outcomes among racial and ethnic groups for patients with a specific kidney complication related to liver disease, known as hepatorenal syndrome–acute kidney injury (HRS‐AKI).
- We found that Black patients faced higher risks of death, partly due to more severe illness and lower socioeconomic status, while Hispanic patients had better survival outcomes.
- These findings highlight the importance of addressing racial and social disparities to potentially improve care quality for patients hospitalized with HRS‐AKI.

## 1 Introduction

About 10% of hospitalised patients with cirrhosis and ascites are afflicted by hepatorenal syndrome–acute kidney injury (HRS‐AKI), a functional form of AKI caused by splanchnic vasodilation and reduced effective arterial volume [1]. The diagnosis carries a poor prognosis, with a median survival of 2 weeks [2]. Definitive treatment for HRS involves liver transplantation (LT), but when unavailable, vasoconstrictors (e.g. midodrine, norepinephrine, terlipressin), albumin and octreotide can be used as temporary adjuncts [1]. Given the involvement of dual organ failure in HRS‐AKI, multidisciplinary care is essential for managing both liver and kidney dysfunction effectively [3, 4].

Racial disparities in health outcomes [5] for Black patients with hepatic or renal diseases are well‐documented, whereas evidence for Hispanic patients is mixed [6, 7, 8]. In those with cirrhosis, Black patients present at more advanced stages of liver disease compared to their white counterparts [9, 10]. They also suffer from higher all‐cause mortality [9, 10], while Hispanics experience lower mortality [11, 12]. Viral hepatitis, a common cause of cirrhosis, remains undertreated among minority populations [8], and Black and Hispanic patients are less likely to undergo LT for portal hypertension‐related complications [10]. In those who suffer from AKI, Black patients experience a worse prognosis [6, 13].

The discordant health outcomes among patients with chronic liver disease are partially related to structural and social determinants of health (SSDOH) or the social policies and structures that mediate access to healthcare, employment, and education [6, 8, 14, 15]. However, the extent of disparity in SSDOH among patients hospitalised with HRS‐AKI has not been well identified for any racial group. In this US nationwide study, we sought to determine whether health outcomes in patients hospitalised with HRS‐AKI differed by racial and ethnic group. Specifically, the aims were to (1) report, by race and ethnicity, demographic and clinical characteristics of patients admitted with HRS‐AKI from 2016 to 2020, (2) examine racial and ethnic variations in mortality and length of stay (LOS) and (3) determine to what extent differences in mortality across groups were attributable to social, economic or clinical factors.

## 2 Methods

We conducted a cross sectional study of patients hospitalised with HRS‐AKI from 2016 to 2020, using the national inpatient sample (NIS) database. The NIS of the Healthcare Cost and Utilisation Project (HCUP) is a national database of discharge data for hospital admissions from non‐federal hospitals in the United States, which reports patient characteristics, length of stay, clinical diagnoses, and hospital‐based procedures and surgeries for inpatient encounters. Characteristics of the hospitals in which patients are admitted can also be found. The NIS annually contains all‐payer data, including approximately 7 million hospital discharges from around 4500 hospitals across most states (47 states in 2016, 48 in 2017–2018, and 49 in 2019–2020). In the NIS sampling dataset, the hospital universe is defined as all hospitals open during any part of the calendar year. This dataset represents a stratified 20% self‐weighted random sample of discharges from all hospitals within the sampling frame. Consequently, the estimates of sample means are identical to those of the weighted (i.e. national) estimates. Detailed information on the NIS can be found on its website [16]. To define hospitalised cirrhotic patients with HRS‐AKI, we included patients with cirrhosis who were admitted with a primary, secondary or discharge diagnosis of HRS and were labelled with having acute kidney injury (AKI), as determined by discharge diagnoses 1–5. HRS‐AKI, as defined by ICD‐10 codes used in the study, is listed in Table S1.

The study cohort was stratified by race/ethnicity, and sociodemographic factors (age, sex, insurance payer, household income and hospital type/region) were also described. The aetiology of liver disease and complications from cirrhosis (e.g. coagulopathy, hepatic encephalopathy and variceal bleeding) were reported. The severity of illness was reported based on the All Patient Refined Diagnosis Related Group (APR‐DRG) severity score [17]; a classification system that categorises patients based on their diagnosis, procedures and severity of illness, measured through clinical factors such as comorbidities, complications and the risk of mortality, to assess hospital resource use and predict patient outcomes, with 1 indicating the least severe disease and 4 indicating the most severe disease. Finally, the mortality and length of stay for each group was characterised. When comparing mortality and LOS across groups, White patients served as the reference group. Because data collected from the NIS database contains publicly available de‐identified patient information, review and approval of the Institutional Review Board from the University of Iowa were not required.

### 2.1 Statistical Analysis

We assessed the bivariate associations between sociodemographic/clinical characteristics and race/ethnicity. Descriptive statistics, including *t*‐tests for continuous variables and chi‐square tests for categorical variables, were used to determine differences by group. *p* values < 0.05 were considered significant.

To identify unique associations of clinical and sociodemographic factors with mortality and LOS, a hierarchical regression analysis was conducted (logistic regression for mortality and Poisson regression for LOS [18]). Several models were used, with each subsequent model adding covariates to the previous. Model 1 included race/ethnicity only; Model 2 added biological factors (age, sex); Model 3 added, to both age and sex, sociodemographic variables (hospital type, median household income for each patient's ZIP code and expected payer); Model 4 added, to both age and sex, the clinical variables (aetiology, APR‐DRG severity score, coagulopathy, hepatic encephalopathy, variceal bleed); and finally, Model 5 included all variables (race/ethnicity, age, sex, and all other sociodemographic and clinical variables). We report the estimates as odds ratios for in‐hospital mortality and incidence rate ratios (RR) for LOS, which are the exponentiated coefficients of the term reflecting race and ethnicity. The RR for length of stay compares the counted days of hospitalisation between groups. For example, an RR of 2 implies that the counted days of hospitalisation (i.e. LOS) are twice as long in one group relative to another.

To further estimate the extent to which the association between race/ethnicity and in‐hospital mortality is accounted for by social, economic and clinical variables, we conducted a mediation analysis, considering the following variables as a potential mediators in each model separately: economic factors [insurance payer: Medicaid vs. others], neighbourhood social factors [median household income for patient's ZIP Code (lowest quantile vs. others)], and clinical severity [APR DRG severity score (4 as extreme loss of function due to comorbidity and complications vs. 1–3)]. We used the Baron‐Kenny method extended by VanderWheel et al. [19] to model exposure‐mediation, and mediator‐outcome models with logistic regression models. Bootstrap were used to estimate the standard errors [20]. Consider the use of the exposure‐mediator model as,

$$\text{logit}\;\left(P\left(\text{mediator}=1\;|\;\text{Race},\text{confounder}\right)\right)=\text{β0}+\text{β1}\times \text{Race}+\beta^{’}2\times \text{confounder}.$$

the mediator‐exposure model as,

$$\begin{array}{cc} \text{logit}\;\left(P\left(\text{Death}=1\;|\;\text{Race},\text{mediator},\text{confounder}\right)\right)= & \text{θ0}+\text{θ1}\times \text{Race}+\text{θ2}\\ & \times \text{mediator}+\theta^{’}3\times \text{confounder}. \end{array}$$

and when the outcome is regressed on the exposure and confounder alone, consider the model as,

$$\text{logit}\;\left(P\left(\text{Death}=1\;|\;\text{Race},\text{confounder}\right)\right)=\text{ϕ0}+\text{ϕ1}\times \text{Race}+\phi^{’}2\times \text{confounder}.$$

these expressions essentially identify θ1 as the direct effect (= effect of the race on outcome other than the mediating factors), θ2β1 as the indirect effect (= effect of the race mediated by mediating factors), and (θ2β1)/ϕ1 as “proportion mediated”, that is, proportion of the effect of race explained by a mediator [21].

The statistical analyses were performed using R software version 4.1 (R Foundation for Statistical Computing, Vienna, Austria) including the mediation analysis which used the R package *CMAverse* [20].

## 3 Results

From 2016 to 2020, 16 377 patients in the NIS sample were hospitalised with HRS‐AKI. Of these, 10 922 (67%) were white; 2701 (16%) were Hispanic; 1486 (9%) were Black; 347 (2%) were Asian/Pacific Islander; 342 (2%) were Native American; and 509 (3%) had another racial identity (or it was unknown). Sociodemographic and clinical features of patients admitted with HRS, stratified by race, are presented in Table 1.

**TABLE 1:** Patient characteristics.

	White (*n* = 10 992)	Black (*n* = 1486)	Hispanic (*n* = 2701)	Asian or Pacific Islander (*n* = 347)	Native American (*n* = 342)	Other (*N* = 509)	*p* value
Age (years)	59.77 (12.58)	58.78 (11.94)	58.06 (12.91)	62.13 (13.68)	50.01 (13.05)	56.78 (14.45)	< 0.001
Female (%)	4110 (37.4)	641 (43.1)	957 (35.4)	140 (40.3)	154 (45.0)	169 (33.2)	< 0.001
Insurance payer							
Medicare	4809 (43.8)	599 (40.3)	1019 (37.7)	159 (45.8)	65 (19.0)	175 (34.4)	< 0.001
Medicaid	2150 (19.6)	426 (28.7)	893 (33.1)	77 (22.2)	175 (51.2)	142 (27.9)	
Private insurance	3215 (29.2)	327 (22.0)	553 (20.5)	94 (27.1)	58 (17.0)	128 (25.1)	
Self‐payer	459 (4.2)	81 (5.5)	170 (6.3)	13 (3.7)	14 (4.1)	46 (9.0)	
No charge	23 (0.2)	2 (0.1)	8 (0.3)	0 (0.0)	0 (0.0)	6 (1.2)	
Other	336 (3.1)	51 (3.4)	58 (2.1)	4 (1.2)	30 (8.8)	12 (2.4)	
Income quartile							
First quartile	2756 (25.1)	783 (52.7)	1070 (39.6)	60 (17.3)	193 (56.4)	126 (24.8)	< 0.001
Second quartile	2995 (27.2)	330 (22.2)	722 (26.7)	67 (19.3)	82 (24.0)	103 (20.2)	
Third quartile	2862 (26.0)	231 (15.5)	583 (21.6)	103 (29.7)	49 (14.3)	143 (28.1)	
Fourth quartile	2379 (21.6)	142 (9.6)	326 (12.1)	117 (33.7)	18 (5.3)	137 (26.9)	
Hospital region							
Northeast	2193 (20.0)	235 (15.8)	265 (9.8)	49 (14.1)	11 (3.2)	144 (28.3)	< 0.001
Midwest	2216 (20.2)	275 (18.5)	176 (6.5)	34 (9.8)	72 (21.1)	60 (11.8)	
South	4343 (39.5)	804 (54.1)	951 (35.2)	60 (17.3)	60 (17.5)	178 (35.0)	
West	2240 (20.4)	172 (11.6)	1309 (48.5)	204 (58.8)	199 (58.2)	127 (25.0)	
Hospital type							
Rural	768 (7.0)	70 (4.7)	42 (1.6)	4 (1.2)	35 (10.2)	5 (1.0)	< 0.001
Urban non‐teaching	2215 (20.2)	216 (14.5)	564 (20.9)	80 (23.1)	37 (10.8)	85 (16.7)	
Urban teaching	8009 (72.9)	1200 (80.8)	2095 (77.6)	263 (75.8)	270 (78.9)	419 (82.3)	
Aetiology of liver disease							
Viral (HCV, HBV)	690 (6.3)	199 (13.4)	225 (8.3)	23 (6.6)	21 (6.1)	37 (7.3)	< 0.001
Alcohol	6011 (54.7)	697 (46.9)	1484 (54.9)	97 (28.0)	266 (77.8)	270 (53.0)	< 0.001
Metabolic	1552 (14.1)	289 (19.6)	328 (12.1)	39 (11.2)	35 (10.2)	47 (9.2)	< 0.001
AIH	110 (1.0)	34 (2.3)	45 (1.7)	8 (2.3)	4 (1.2)	14 (2.8)	< 0.001
Cholestatic (PBC or PSC)	103 (0.9)	13 (0.9)	20 (0.7)	4 (1.2)	1 (0.3)	6 (1.2)	0.69
APR‐DRG severity score							
2	85 (0.8)	9 (0.6)	16 (0.6)	0 (0.0)	2 (0.6)	4 (0.8)	0.01
3	5265 (47.9)	665 (44.8)	1205 (44.6)	149 (42.9)	169 (49.4)	224 (44.0)	
4	5642 (51.3)	812 (54.6)	1480 (54.8)	198 (57.1)	171 (50.0)	281 (55.2)	
Clinical features							
Coagulopathy hepatic	1512 (13.8)	214 (14.4)	492 (18.2)	71 (20.5)	58 (17.0)	84 (16.5)	< 0.001
Encephalopathy	117 (1.1)	20 (1.3)	29 (1.1)	9 (2.6)	2 (0.6)	6 (1.2)	0.119
Variceal bleed	60 (0.5)	12 (0.8)	20 (0.7)	3 (0.9)	1 (0.3)	3 (0.6)	0.634

With respect to age, on average, Asian/Pacific Islander patients were oldest at the time of admission [62 (SD: 14) years], while Native American patients were the youngest [50 (SD: 13) years]. The proportion of females was highest among Native American patients but lowest among Hispanic patients (45% vs. 35%, respectively, *p* < 0.001). The economic status of patients admitted with HRS also varied among groups (*p* < 0.001), with Black patients and Native American patients having the greatest proportion of patients in the lowest income bracket (53% and 56%, respectively). Meanwhile, Asian/Pacific Islander patients had the highest fraction (33%) in the top income bracket. Group composition also differed by geographic area (*p* < 0.001). The greatest proportion of white patients and Black patients was observed in the South, while Hispanic, Asian/Pacific Islander, and Native‐American patients tended to have the greatest representation in the West. Most patients with HRS were hospitalised in urban teaching hospitals. Native American patients represented the highest fraction (10%) who were admitted to rural hospitals.

The aetiology of cirrhosis differed among the groups (*p* < 0.001), although alcohol use, metabolic disease or viral hepatitis (in descending order of frequency) predominated as causes in each group. When APR‐DRG severity scores were compared, Asian/Pacific Islander patients had the highest fraction (57%) of those of with a maximum score of 4 (indicating the most severe disease), while Native American patients represented the lowest fraction (50%) in this category. Rates of hepatic encephalopathy and variceal bleed did not vary between groups, though coagulopathy was seen most often in Asian/Pacific Islander patients (21%) and least frequently among white patients (14%, *p* = 0.01).

The associations between race/ethnicity and both mortality and LOS are shown in Table 2. Black patients demonstrated a significantly higher odds of death [OR 1.13–1.20, 95% confidence interval (CI) > 1], compared to their white counterparts, even after adjusting for [1] sex and age, [2] sex, age and sociodemographic variables, and [3] sex, age and APR‐DRG severity. However, the association was not statistically significant when all variables were included (i.e. Model 5). Compared to white patients, Hispanic patients tended to have lower odds of death [OR: 0.86 (95% CI: 0.77–0.97)], and the statistical significance was maintained throughout Models 1–5. Other groups did not demonstrate a significant mortality benefit or harm when compared to white patients. In the analysis of LOS, Black patients with HRS‐AKI had longer LOS, even after adjusting for covariates, while Hispanic patients demonstrated longer LOS only in Model 1, which included only race/ethnicity, and in Model 2, which adjusted for age and sex.

**TABLE 2:** Hierarchical logistic and Poisson regression models to estimate the association of race with mortality and length of hospital stay, adjusted for biological, sociodemographic and/or clinical variables (White as a reference group).

	Model 1 (unadjusted)	Model 2 (sex/age)	Model 3 (sex/age/sociodemographic variables^a^)	Model 4 (sex/age/clinical variables^b^)	Model 5 (full model with all variables)
Mortality	OR (95% CI)				
White	(ref)	(ref)	(ref)	(ref)	(ref)
Black	1.19 (1.03, 1.36)	1.20 (1.04, 1.38)	1.18 (1.02, 1.36)	1.16 (1.01, 1.34)	1.13 (0.90, 1.31)
Hispanic	0.86 (0.77, 0.97)	0.87 (0.78, 0.98)	0.81 (0.72, 0.92)	0.83 (0.73, 0.93)	0.78 (0.68, 0.88)
Asian/Pacific Islander	1.30 (0.99, 1.69)	1.28 (0.98, 1.67)	1.21 (0.92, 1.57)	1.17 (0.89, 1.53)	1.14 (0.85, 1.49)
Native Americans	0.99 (0.74, 1.32)	1.07 (0.79, 1.43)	0.99 (0.73, 1.32)	1.13 (0.83, 1.51)	1.04 (0.76, 1.40)
Others	1.19 (0.95, 1.49)	1.22 (0.97, 1.52)	1.15 (0.91, 1.41)	1.17 (0.93, 1.51)	1.11 (0.88, 1.40)
LOS	RR (95% CI)				
White	(ref)	(ref)	(ref)	(ref)	(ref)
Black	1.10 (1.08, 1.12)	1.09 (1.07, 1.11)	1.08 (1.06, 1.10)	1.07 (1.05, 1.09)	1.06 (1.04, 1.08)
Hispanic	1.04 (1.03, 1.06)	1.03 (1.02, 1.05)	1.02 (1.00, 1.03)	1.01 (1.00, 1.02)	1.00 (0.99, 1.02)
Asian/Pacific Islander	1.02 (0.98, 1.06)	1.04 (1.02, 1.06)	0.95 (0.92, 0.99)	0.99 (0.96, 1.03)	0.98 (0.94, 1.01)
Native Americans	1.01 (0.98, 1.05)	0.93 (0.90, 0.96)	0.95 (0.92, 0.99)	0.94 (0.91, 0.98)	0.97 (0.94, 1.01)
Others	1.22 (1.19, 1.32)	1.20 (1.16, 1.23)	1.14 (1.11, 1.17)	1.17 (1.14, 1.21)	1.12 (1.09, 1.15)

^a^ Hospital type, median household income for patient's ZIP Code, expected payer.

^b^ Aetiology, APR‐DGR severity, Coagulopathy, Hepatic encephalopathy, Variceal bleed.

Table 3 summarised the result of the mediation analysis to evaluate the magnitude of social determinants of health and clinical severity on the racial disparity on in‐hospital mortality. Higher mortality rates experienced by Black patients with HRS‐AKI were largely mediated both by clinical and neighbourhood income [proportion mediated (PM): 0.19 (95% CI 1.189–1.192) and 0.17 (0.16–0.18), respectively], and to a lesser extent, by insurance payer (PM: 0.02). However, the survival advantage of Hispanic patients was not explained by any economic, social or clinical factors, evidenced by the negative direct effects (ORs < 1 with 95% CIs not crossing 1) and negative proportions mediated. The mediation analysis for other racial groups, including Asian/Pacific Islanders and Native Americans, yielded non‐significant results.

**TABLE 3:** Mediation analysis to evaluate the effect of social determinants of health and clinical severity on the racial disparity on in‐hospital mortality.

Mediator	Effect type	Black	Hispanic	Asian /Pacific Islander	Native American	Other
		Estimate [95% CI]	Estimate [95% CI]	Estimate [95% CI]	Estimate [95% CI]	Estimate [95% CI]
Economic factor (payer: Medicaid vs. others [ref])	Direct effect (OR^a^)	1.14 [0.99 to 1.1]	0.81 [0.79 to 0.83]	1.03 [0.96 to 1.10]	1.01 [0.95 to 1.07]	1.04 [0.97 to 1.12]
	Indirect effect (OR^a^)	1.002 [1.001 to 1.003]	1.01 [1.004 to 1.02]	1.00 [0.999 to 1.002]	1.01 [0.999 to 1.014]	1.02 [0.998 to 1.015]
	Proportion mediated	0.02 [0.02 to 0.02]	−0.05 [−0.049 to −0.051]	0.014 [−0.15 to 0.15]	0.38 [−3.1 to 2.1]	0.020 [−0.16 to 0.16]
Neighbourhood social factor (Median household income for patient's ZIP Code: lowest quantile vs. others [ref])	Direct effect (OR^a^)	1.14 [0.94 to 1.38]	0.80 [0.73 to 0.88]	1.03 [0.95 to 1.09]	1.01 [0.95 to 1.06]	1.03 [0.96 to 1.10]
	Indirect effect (OR^a^)	1.03 [1.02 to 1.04]	1.01 [1.006 to 1.02]	0.997 [0.996 to 1.001]	1.001 [0.999 to 1.003]	1.002 [0.996 to 1.005]
	Proportion mediated	0.17 [0.16 to 0.18]	−0.04 [−0.039 to −0.041]	−0.11 [−0.50 to 0.94]	0.067 [−0.53 to 0.54]	0.11 [−0.62 to 0.98]
Clinical severity (DRG severity score: 4, extreme loss of function vs. 1–3 [ref])	Direct effect (OR^a^)	1.14 [1.1 to 1.19]	0.80 [0.77 to 0.83]	1.04 [0.98 to 1.11]	1.01 [0.94 to 1.07]	1.03 [0.99 to 1.12]
	Indirect effect (OR^a^)	1.03 [1.02 to 1.04]	1.03 [1.029 to 1.032]	1.02 [0.99 to 1.03]	0.99 [097 to 1.01]	1.01 [0.96 to 1.04]
	Proportion mediated	0.19 [0.1885 to 0.1915]	−0.13 [−0.129 to −0.131]	0.28 [−0.34 to 1.98]	−0.43 [−0.70 to 0.52]	0.29 [−0.43 to 2.27]

^a^ Odds ratio.

## 4 Discussion

In this nationwide US study, we examined characteristics of patients admitted with HRS‐AKI and evaluated racial disparities in mortality and length of stay. HRS‐AKI is distinct due to the involvement of both liver and kidney dysfunction, making its treatment more complex than that of other renal or hepatic diseases [1]. Managing HRS‐AKI requires coordinated care from hepatology, nephrology and critical care [3, 4], which can highlight disparities in access to specialised care. Racial differences in accessing these services, along with timely interventions like vasoconstrictors and liver transplantation, likely contribute to worse outcomes for certain groups [9, 10]. Given past reports of racial disparities in renal and hepatic disorders [6, 7, 8] (which have been studied separately), we anticipated similar, or possibly even more pronounced, disparities in patients with this dual organ dysfunction.

When clinical outcomes were analysed, we found that Black patients with HRS‐AKI faced higher odds of death and longer LOS even after adjusting for [1] sex and age, [2] sex, age and sociodemographic variables, and [3] sex, age and APR‐DRG severity. Once all covariates (age, sex, sociodemographic variables, aetiology of liver disease, APR‐DRG severity score, and status of hepatic encephalopathy, variceal bleed and coagulopathy) were included, however, no significant difference in mortality rates was seen. Our results align with those of a prior nationwide study. Although HRS was not specifically studied at the time, Nguyen et al. confirmed that Black patients with complications related to portal hypertension (e.g. ascites, spontaneous bacterial peritonitis, hepatic encephalopathy, variceal bleeding) have a significantly increased odds of death compared to White patients [22]. Based on the results from our mediation analysis, clinical severity contributed to the mortality difference, as expected, but so too did neighbourhood social factors.

Social determinants of health influence individuals' opportunities, resources, socioeconomic status, vocation, as well as their access to and outcomes of healthcare (Table S2 summarises racial disparities in general health, as well as hepatology‐ and nephrology‐related health outcomes.) [8]. Limitations to hepatology care may have caused some Black patients to seek care later than they would have otherwise, leading to high mortality rates in‐hospital, particularly if they develop complications related to portal hypertension, like HRS‐AKI [8]. Structural racism in eGFR equations, using race‐based coefficients, has delayed both AKI and CKD diagnoses in Black patients, often leading to underdiagnosis in early stages and higher rates of kidney failure, and limited access to diagnostic technologies like biomarker testing in underserved communities further exacerbates inequities in disease assessment and progression risk [7]. Most (70%) patients with cirrhosis are unaware about their condition, and rates of unawareness are higher among Black patients [23]. Though the reason behind increased LOS among Black patients with HRS‐AKI remains speculative, delayed presentation and/or lack of prior care are potential explanations.

On the other hand, out study showed Hispanics with HRS‐AKI experienced lower mortality rates compared to white patients, even after adjusting for clinical and demographic variables. Differences in clinical severity, social determinants or economic factors failed to explain the paradox. The survival advantage among Hispanics with cirrhosis or other health conditions, particularly related to cardiovascular and renal disease, has been reported before and has been termed the “Hispanic paradox.” [11, 12] Several theories have been put forth to account for these findings, including (1) the “healthy migrant” theory, which suggests that Hispanics who immigrate to the United States may be healthier than the American‐born population (2) the “salmon” bias theory, which suggests that Hispanics return to their home countries for support when their illness worsens and (3) and lifestyle/social factor theory, which suggests that diet, culture and strong familial structure may positively influence health outcomes [12].

In our study, the mean age at admission for White patients with HRS‐AKI was 60 years. In comparison, Asian/Pacific Islander patients had the oldest average age at the time of admission (62 years), while Native American patients' average age (50 years) was the youngest. HRS‐AKI usually presents in the sixth or seventh decade of life [22]. To our knowledge, differences in age at admission across racial and ethnic groups have not previously been reported. Given the high morbidity and mortality associated with HRS‐AKI, it may be that Native American patients with cirrhosis are decompensating (e.g. developing ascites, a necessary precursor for HRS‐AKI) sooner than their counterparts and suffering adverse renal consequences as a result. Alternatively, Native American patients with decompensated cirrhosis may, at younger ages, be prone to risk factors for developing HRS‐AKI, such as infection (e.g. bacterial translocation, spontaneous bacterial peritonitis, etc.), cirrhotic cardiomyopathy and systemic inflammation [1]; or, they may have lacked access to hepatology care prior to hospitalisation [24]. It is unclear why Native American patients had the highest proportion of females (45%) admitted with HRS‐AKI. The relatively narrow sex gap among Native American patients with HRS‐AKI warrants further research. With respect to clinical characteristics, we found that Asian/Pacific Islander patients hospitalised with HRS‐AKI exhibited the most severe illness, as they held the greatest fraction of patients (57%) with APR‐DRG severity score of 4. Asian/Pacific Islander patients with HRS‐AKI also had the highest proportion (21%) with coagulopathy, implying that they suffered from greater hepatic dysfunction, in turn fuelling their renal dysfunction. Of all the groups in the study, Asian/Pacific Islander patients were admitted at the oldest average age; this may have further contributed to their high clinical severity scores.

There were several strengths of this study. First, it was a nationwide study with a large sample size, such that several racial and ethnic groups could be represented. Second, both HRS and AKI were used as inclusion criteria in the ICD‐10 coding system to ensure that our cohort was accurately diagnosed. Third, we used rigorous analyses with different models to account for covariates. Last, we used a mediation analysis to determine the underlying cause of mortality differences among Black and Hispanic patients. The estimates from multiple regression models, including apparent confounders examining racial disparities, were consistent, supporting the robustness of our analysis. However, we acknowledge that potential residual confounding, both clinically and socioeconomically, may still exist. Other limitations of the study include those inherent to any research using the National Inpatient Sample, including potential mislabelling or misclassification of patients' clinical or demographic characteristics. Patients with adcanced end‐stage liver disease, who are not expected to have an improved prognosis and do not receive aggressive medical intervention, may have been selectively excluded from or mislabelled in our target population. However, given the large sample size, much smaller proportion of such cases should not significantly affect the results, and our target population consists of those actively seeking medical management. Also, granular information regarding each case (e.g. detailed clinical course, laboratory or radiological test results, provoking factors of HRS‐AKI, severity of cirrhosis/portal hypertension complications, or treatment) was not included in the database. Lastly, although the NIS employs a self‐weighted design to provide national estimates, there remains the possibility of biased sampling among hospitals due to the inherent nature of sampling from the overall hospital population.

In summary, this nationwide US study demonstrated that, partly due to clinical and neighbourhood social factors, Black patients with HRS‐AKI suffer from higher mortality, compared to White patients. On the other hand, Hispanic patients with HRS‐AKI have a survival advantage despite their lower socioeconomic status and less favourable clinical presentation. Native American patients with HRS‐AKI tend to be hospitalised at younger ages, while Asian/Pacific Islander patients with HRS‐AKI are hospitalised at older ages and more often suffer from severe disease. The differences in clinical characteristics, demographics, and outcomes among various racial and ethnic groups with HRS‐AKI demands not only further study, but also increased awareness; this will ensure that attention is focused on patients with HRS‐AKI who are most vulnerable.

## Conflicts of Interest

The authors declare no conflicts of interest.

## Supporting information


Table S1.

Table S2.

## Data Availability

This study used third party data made available under licence that the author does not have permission to share. Requests to access the data should be directed to AHRQ/HCUP at https://hcup‐us.ahrq.gov/tech_assist/centdist.jsp.
