# Supplementary material for: The Association of Race With Outcomes in Hospitalised Patients With Hepatorenal Syndrome: Nationwide Cohort Study
Source: Liver Int. 2024 Dec 25;45(1):e16226. doi: 10.1111/liv.16226 (PMC11669052; doi:10.1111/liv.16226)
Supplement: Supplementary file 1 — Table S1. [file LIV-45-0-s002.docx]

**Table S1.** ICD-10 codes used to identify HRS-AKI*

|  | ICD-10 |
| --- | --- |
| Hepatorenal syndrome | K76.7 |
| Acute renal failure | N17.9 |
| Liver cirrhosis | K70.3, K70.30, K70.31, K74.3, K74.4, K74.5, K74.6, K74.60, K74.69, K76.6, K76.7, K76.81, K72.1, K72.10, K72.11, I85.0, I85.00, I85.01, C22.0, C22.8, K65.2, K72.10, K72.11, I86.4, I85.01, I85.11. |

* The diagnosis of HRS-AKI was identified by codes for hepatorenal syndrome or acute renal failure, in conjunction with any codes related to liver cirrhosis.
