# Supplementary material for: The Association of Race With Outcomes in Hospitalised Patients With Hepatorenal Syndrome: Nationwide Cohort Study
Source: Liver Int. 2024 Dec 25;45(1):e16226. doi: 10.1111/liv.16226 (PMC11669052; doi:10.1111/liv.16226)
Supplement: Supplementary file 2 — Table S2. [file LIV-45-0-s001.docx]

# Table S2. Racial Disparities in Healthcare

| **Racial disparities in healthcare**^1^ | | |
| --- | --- | --- |
| **Domain** | **Details** | **Related health outcomes** |
| Economic Stability | Employment, Income, Expenses, Debt, Medical Bills, Support | Mortality, Morbidity, Life Expectancy, Health Care Expenditures, Health Status, Functional Limitations |
| Neighborhood and Physical Environment | Housing, Transportation, Parks, Playgrounds, Walkability, Zip Code/Geography |  |
| Education | Literacy, Language, Early Childhood Education, Vocational Training, Higher Education |  |
| Food | Food Security, Access to Healthy Options |  |
| Community, Safety, & Social Context | Social Integration, Support Systems, Community Engagement, Stress, Exposure to Violence/Trauma, Policing/Justice Policy |  |
| Health Care System | Health Coverage, Provider & Pharmacy Availability, Access to Linguistically and Culturally Appropriate & Respectful Care, Quality of Care |  |
| **Disease specific racial disparity in healthcare related to Liver and Kidney disease** | | |
| **Disease** | **Details** | |
| **Liver disease**^2^ | (1) Disease Burden  - **Alcohol-Associated Liver Disease (ALD)**: Higher prevalence in Hispanic and Black populations compared to White populations due to factors such as alcohol outlet density and multigenerational effects of zoning and redlining. - **Nonalcoholic Fatty Liver Disease (NAFLD)**: Hispanic persons are disproportionately affected, with the highest prevalence of NAFLD and associated advanced fibrosis. - **Chronic Hepatitis B (CHB)**: Asian individuals (2.74%) and Black individuals (0.64%) bear a higher prevalence compared to other groups; most are foreign-born. - **Hepatitis C Virus (HCV)**: The highest acute incidence is in American Indian and Alaskan Native (AI/AN) populations, while chronic incidence is highest among Black populations. - **Socioeconomic Factors**: Food insecurity is strongly associated with advanced liver fibrosis in NAFLD, independent of poverty and education.  (2) Access to Care  - **Alcohol Use Disorder (AUD)**: Racial minorities like Black and Hispanic individuals have lower access to specialty AUD treatment due to socioeconomic and systemic barriers. - **HCV Treatment**: Black and Hispanic individuals face lower screening and treatment rates for HCV compared to White individuals, compounded by Medicaid and insurance barriers. - **LT for Alcoholic Hepatitis**: Black patients and women are underrepresented in liver transplantation for acute alcoholic hepatitis despite higher disease burden. - **HBV Vaccination and Screening**: Socioeconomically disadvantaged groups have lower rates of vaccination-induced immunity and screening, particularly among foreign-born populations. - **Clinical Trial Participation**: Hispanic persons remain underrepresented in clinical trials for NASH and other liver diseases despite the high burden of disease. - Cirrhosis/end-stage liver disease: Black and Hispanic patients remained less likely to receive life-saving procedures for complications due to cirrhosis, including liver transplants  (3) Outcomes  - **Mortality in ALD**: Native American women and White women showed the most significant absolute increases in ALD mortality in recent years. - **NAFLD Hospitalizations**: Black patients have lower hospitalization rates but longer stays and lower discharge-to-home rates compared to White patients. - **HCV Mortality**: Black individuals experience the highest HCC-related mortality, driven by untreated HCV. - **HBV Outcomes**: African immigrants with CHB often present with advanced-stage disease and experience significantly higher mortality rates. - **Autoimmune liver disease**: Black patients with autoimmune liver disease face higher inpatient mortality compared to other groups. | |
| **Kidney disease**^3^ | (1) Disease Burden  - Black individuals in the U.S. have a 2.6-fold higher risk of kidney failure requiring dialysis or transplantation compared to White individuals. - Hispanic populations are 1.5 times more likely to develop kidney failure than their White counterparts. - Neighborhood segregation, food insecurity, and exposure to pollutants disproportionately increase CKD risk in marginalized communities. - Comorbidities such as diabetes and hypertension, major drivers of CKD, are more prevalent in Black and Hispanic populations. - Higher allostatic load among Black individuals indicates chronic stress contributing to faster CKD progression.  (2) Diagnosis  - Structural racism in eGFR equations, using race-based coefficients, has delayed CKD diagnosis in Black patients. - Black patients often face underdiagnosis of CKD in its early stages, leading to higher rates of kidney failure. - eGFR disparities due to presumed higher muscle mass in Black individuals perpetuate diagnostic inequities. - Race-based algorithms obscure individual health factors, misrepresenting disease severity and progression risks. - Access to advanced diagnostic technologies, such as biomarker testing (e.g., cystatin C), remains limited in underserved communities.  (3) Access to Care  - Black individuals are less likely to receive nephrology care prior to initiating dialysis compared to White individuals. - They are more likely to start dialysis using a catheter instead of an arteriovenous fistula, increasing infection risks. - Structural barriers, including implicit bias and complicated evaluation processes, reduce access to kidney transplantation. - Insurance limitations disproportionately affect low-income racial minorities, reducing access to dialysis and transplant services. - Home dialysis modalities, associated with better outcomes, are underutilized among Black patients due to socioeconomic disparities.  (4) Outcomes  - Black patients with CKD experience more rapid disease progression and worse survival rates than White patients. - Disparities in pre-transplant care lead to lower rates of successful kidney transplantation among Black individuals. - CKD-related cardiovascular complications are more prevalent in racial and ethnic minorities. - Stress-related biological factors, such as sympathetic nervous system activation, exacerbate CKD severity in marginalized groups. - Reduced access to early intervention results in higher mortality rates for advanced CKD in Black and Hispanic populations. | |

**Reference:**

1. <https://www.kff.org/racial-equity-and-health-policy/dashboard/racial-equity-and-health-data-dashboard/>. Accessed on 11/25/2024;

2. Kardashian A, Serper M, Terrault N, Nephew LD. Health disparities in chronic liver disease. *Hepatology*. Apr 1 2023;77(4):1382-1403. doi:10.1002/hep.32743

3. Eneanya ND, Boulware LE, Tsai J, et al. Health inequities and the inappropriate use of race in nephrology. *Nat Rev Nephrol*. Feb 2022;18(2):84-94. doi:10.1038/s41581-021-00501-8
